# An Unmethylated 3′ Promoter-Proximal Region Is Required for Efficient Transcription Initiation

**DOI:** 10.1371/journal.pgen.0030027

**Published:** 2007-02-16

**Authors:** Ruth Appanah, David R Dickerson, Preeti Goyal, Mark Groudine, Matthew C Lorincz

**Affiliations:** 1 Department of Medical Genetics, The University of British Columbia, Vancouver, British Columbia, Canada; 2 Division of Basic Sciences, Fred Hutchinson Cancer Research Center, Seattle, Washington, United States of America; University of Cambridge, United Kingdom; 3 Department of Radiation Oncology, University of Washington School of Medicine, Seattle, Washington, United States of America

## Abstract

The promoter regions of approximately 40% of genes in the human genome are embedded in CpG islands, CpG-rich regions that frequently extend on the order of one kb 3′ of the transcription start site (TSS) region. CpGs 3′ of the TSS of actively transcribed CpG island promoters typically remain methylation-free, indicating that maintaining promoter-proximal CpGs in an unmethylated state may be important for efficient transcription. Here we utilize recombinase-mediated cassette exchange to introduce a Moloney Murine Leukemia Virus (MoMuLV)-based reporter, in vitro methylated 1 kb downstream of the TSS, into a defined genomic site. In a subset of clones, methylation spreads to within ∼320 bp of the TSS, yielding a dramatic decrease in transcript level, even though the promoter/TSS region remains unmethylated. Chromatin immunoprecipitation analyses reveal that such promoter-proximal methylation results in loss of RNA polymerase II and TATA-box-binding protein (TBP) binding in the promoter region, suggesting that repression occurs at the level of transcription initiation. While DNA methylation-dependent trimethylation of H3 lysine (K)9 is confined to the intragenic methylated region, the promoter and downstream regions are hypo-acetylated on H3K9/K14. Furthermore, DNase I hypersensitivity and methylase-based single promoter analysis (M-SPA) experiments reveal that a nucleosome is positioned over the unmethylated TATA-box in these clones, indicating that dense DNA methylation downstream of the promoter region is sufficient to alter the chromatin structure of an unmethylated promoter. Based on these observations, we propose that a DNA methylation-free region extending several hundred bases downstream of the TSS may be a prerequisite for efficient transcription initiation. This model provides a biochemical explanation for the typical positioning of TSSs well upstream of the 3′ end of the CpG islands in which they are embedded.

## Author Summary

Genes, the functional units of heredity, are made up of DNA, which is packaged inside the nuclei of eukaryotic cells in association with a number of proteins in a structure called chromatin. In order for transcription, the process of transferring genetic information from DNA to RNA, to take place, chromatin must be decondensed to allow the transcription machinery to bind the genes that are to be transcribed. In mammals, promoters, the starting position of genes, are frequently embedded in “CpG islands,” regions with a relatively high density of the CpG dinucleotide. Paradoxically, while cytosines in the context of the CpG dinucleotide are generally methylated, CpGs flanking the start sites of genes typically remain methylation-free. As CpG methylation is associated with condensed chromatin, it is generally believed that promoter regions must remain free of methylation to allow for binding of the transcription machinery. Here, using a novel method for introducing methylated DNA into a defined genomic site, we demonstrate that DNA methylation in the promoter-proximal region of a gene is sufficient to block transcription via the generation of a chromatin structure that inhibits binding of the transcription machinery. Thus, methylation may inhibit transcription even when present outside the promoter region.

## Introduction

DNA methylation is essential for mammalian development [1,2], playing an important role in maintaining transcriptional silencing of genes on the inactive X chromosome, imprinted genes, and parasitic elements [3,4]. In mammals, DNA methylation occurs predominantly on cytosines in the context of the 5′-CpG-3′ dinucleotide (^m^CpG), and this epigenetic mark is propagated on both parent and nascent strands after DNA replication. The CpG dinucleotide is generally found at a lower than expected frequency in the mammalian genome, with the exception of G + C-rich regions known as CpG islands, which have the statistically expected frequency of CpGs [5]. Analysis of the distribution of DNA methylation reveals that while the majority of cytosines in the context of the CpG dinucleotide are methylated in normal adult somatic tissues, promoter regions containing a high concentration of CpGs, which encompass approximately 70% of mammalian genes [6], typically remain methylation-free [7]. Surprisingly, the relatively high CpG density associated with CpG island promoters frequently extends ∼400–1,000 bp downstream of the transcription start sites **(**TSS) of such genes [6,8], indicating that an unmethylated region extending 3′ of the TSS may be required for efficient transcription.

While it is clear that methylation of promoter regions, including that of the Moloney Murine Leukemia Virus (MoMuLV) [9], leads to silencing at the level of transcription initiation [4,10,11], several lines of evidence suggest that DNA methylation in the promoter proximal region 3′ of the TSS can also have an adverse affect on transcription. Methylation exclusively in the coding region of an episomal reporter for example, yields an ∼10-fold reduction in expression, relative to an unmethylated control [12]. Similarly, transient transfection of reporter constructs methylated in vitro in regions exclusive of the promoter yields a dramatic decrease in expression level relative to unmethylated controls [13,14]. Furthermore, microinjection experiments of mammalian cells [15] or *Xenopus* oocytes [16] with in vitro methylated reporter constructs reveals that dense methylation 3′ of an unmethylated promoter can dramatically decrease expression level, particularly when located in close proximity to the promoter.

Using the Cre/*loxP*-based recombination system, recombinase-mediated cassette exchange (RMCE) [11,17,18], we recently showed that a region of dense methylation located ∼1 kb downstream of the TSS of a *p16*/*CDKN2A* CpG island promoter attenuates expression level by decreasing elongation efficiency [19]. Taken together, these results reveal that methylation 3′ of the TSS may adversely affect the efficiency of transcription. However, a detailed analysis of the interplay between proximity of DNA methylation to the TSS, transcription efficiency, and chromatin structure has not yet been reported.

Here we used RMCE to target a MoMuLV long terminal repeat (LTR)-based transgene encoding “humanized” green fluorescent protein (GFP), either unmethylated or methylated in vitro exclusively in a region 3′ of the TSS, to a specific intergenic genomic site in murine erythroleukemia (MEL) cells. We show that the methylation pattern introduced in vitro is unstable in vivo, with spreading of methylation towards the TSS occurring in a subset of the clones isolated. When methylation spreads to within ∼320 bp 3′ of the TSS, expression is dramatically reduced, relative to clones bearing an unmethylated cassette integrated at the same site. Surprisingly, such promoter-proximal methylation inhibits RNA polymerase II (RNAPII) recruitment and TATA-box-binding protein (TBP) binding at the unmethylated promoter. Analysis of the modification state of the amino-terminal tail of H3 reveals that while H3 trimethylated on lysine 9 (H3K9me3) is confined to the patch-methylated region, the unmethylated promoter is hypo-acetylated at this residue, and nucleosome positioning is dramatically altered around the TSS. Based on these observations, we propose that while methylation ∼1,000 bp 3′ of the TSS has a relatively modest effect on transcription elongation, methylation ∼300 bp 3′ of the TSS generates a chromatin structure that precludes efficient transcription initiation from a methylation-free promoter.

## Results

**Figure 1:**
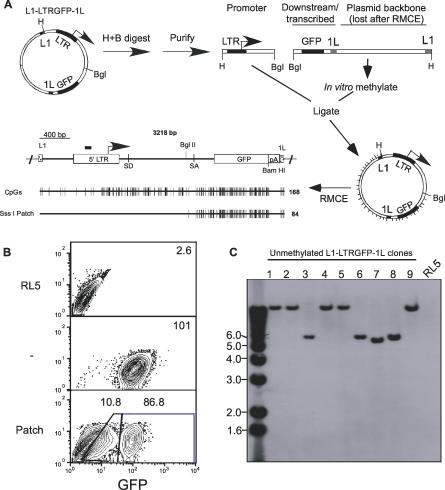
Generation and Targeting of the Patch-Methylated Reporter Cassette (A) The construct L1-LTRGFP-1L was digested with HindIII (H) and BglII (Bgl), generating two fragments, one containing the LTR and ∼1 kb of the transcription unit and the other containing the 3′ end of the transcription unit, which includes the GFP gene. The latter fragment was methylated in vitro and ligated to the unmethylated promoter fragment, regenerating the original plasmid. RL5 MEL cells were cotransfected with this “patch”-methylated plasmid or an unmethylated control in combination with the Cre-expression plasmid CMV-Cre. Successful recombination between the *loxP* sites flanking the LTRGFP transgene and the pre-existing HyTK cassette yielded a targeted L1-LTRGFP-1L cassette initially methylated at the CpG sites as shown (L1 and 1L, *loxP* sites; SD, splice donor; SA, splice acceptor; pA, SV40 polyA). (B) FACS analyses of the RL5 parent line and ganciclovir resistant “pools” harboring the unmethylated (−) or patch-methylated L1-LTRGFP-1L cassette are shown as 5% probability plots. Median fluorescence values for each are shown, with the GFP+ patch-methylated population divided into two populations via gating, as shown. (C) Southern analysis of genomic DNA isolated from clones harboring the unmethylated L1-LTRGFP-1L cassette was conducted using a GFP probe. With the exception of clone 7, all clones harbor the cassette integrated in the RL5 site in one of two possible orientations.

To determine if DNA methylation exclusive of the promoter/TSS region of a CpG island promoter influences transcription initiation, we introduced a transgene containing the MoMuLV LTR driving expression of GFP, either unmethylated or “patch” methylated in vitro exclusively in a region ∼1 kb 3′ of the TSS (Figure 1A), into the RL5 integration site in MEL cells by RMCE [11,17]. This integration site was recently cloned and mapped to the intergenic region between the *Tal1* and *Map17* genes on Chromosome 4 [20]. Flow cytometric analysis of the pool of ganciclovir resistant cells electroporated with the control unmethylated (−) cassette revealed a high and homogeneous level of GFP expression (Figure 1B). In contrast, analysis of the pool of ganciclovir resistant cells harboring the patch-methylated cassette revealed heterogeneous GFP expression, with one population expressing at a level approaching that of the unmethylated cassette and another expressing at a relatively low level. To study the transcriptionally active subpopulations in greater detail, GFP+ cells were sorted, and subclones generated. As expected, the majority of clones generated with the unmethylated cassette harbor the transgene at the RL5 site in one of two possible orientations, as determined by Southern blotting (Figure 1C). Thus, consistent with our previous work, these data reveal that an unmethylated cassette is expressed at relatively high levels, irrespective of genomic orientation. In all subsequent experiments, control clones harboring an “orientation-matched” unmethylated cassette were analyzed in parallel with clones harboring the patch-methylated cassette.

**Figure 2:**
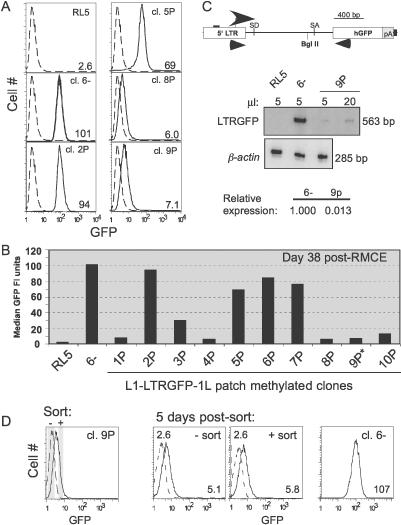
The Patch-Methylated L1-LTRGFP-1L Cassette is Expressed at a Low Level in a Subset of Clones (A) Orientation-matched, unmethylated (−) and patch-methylated (P) clones were analyzed for GFP expression by flow cytometry along with the RL5 parent cell line (dashed line), which is under-laid with each clone for reference. Median fluorescence values are displayed in the lower right hand corner of each histogram. (B) The median GFP fluorescence values of RL5, clone 6− and 10 patch-methylated clones analyzed at day 38 post-RMCE are shown. Clone 9P (marked with an asterisk) was chosen for further study. (C) Quantitative duplex RT-PCR analysis of total RNA isolated from a representative low-expressing patch-methylated clone (9P), the unmethylated control clone 6−, and the RL5 parent line was conducted using primers (horizontal arrows) specific for the spliced transgenic mRNA and the endogenous *β-actin* gene. Relative expression level was determined by normalizing the transgene-specific amplification product to the *β-actin* gene product. (D) Two sets of electronic gates were applied to sort relatively low- and high-expressing cells from clone 9P. A total of 10^4^ viable cells of each subpopulation were sorted and cultured for five days prior to reanalysis by flow cytometry. The median fluorescence values for each sorted pool are shown in the lower right hand corner of each histogram, along with that of the RL5 parent line, and clone 6− for reference.

To determine whether the heterogeneity in expression detected in the pool of cells harboring the patch-methylated cassette reflects the presence of cells with distinct, stable expression states, clones were analyzed by flow cytometry at day 38 post-electroporation (Figure 2A and 2B). Patch-methylated clones (identified with an affixed “P” throughout the remainder of this article) of two distinct classes were detected; one expressing GFP at levels close to the unmethylated cassette and another expressing at significantly reduced levels. Comparison of the median GFP fluorescence values of ten clones harboring the patch-methylated cassette reveals that the “dull” clones express at a level ∼2%–10% of the unmethylated control (with the exception of clone 3P, which shows a heterogeneous pattern of expression; unpublished data), while the “bright” clones express at a level approaching that of the unmethylated control (Figure 2B). To independently determine the relative expression level of the “low-expressing” class of clones, the steady state level of mRNA of an unmethylated control (6−), and a representative low-expressing patch-methylated clone (9P) was determined by RT-PCR (Figure 2C). Consistent with the flow cytometry results, the methylated clone was found to express mRNA at ∼2% of the level of the unmethylated clone. To confirm that the clonal populations showing a low level of expression do not include transcriptionally silent cells, subpopulations of clone 9P cells showing relatively high (above the 98th percentile of the GFP negative parent line) or low (indistinguishable from the parent line) expression were sorted, cultured for 5 d, and reanalyzed by flow cytometry (Figure 2D). The expression profiles of these populations were very similar, indicating that the cells in this clone are homogeneous with respect to expression level. Taken together, these results reveal that a subset of patch-methylated clones harbor a cassette from which expression is dramatically reduced relative to an unmethylated control cassette integrated at the same site in the same orientation.

**Figure 3:**
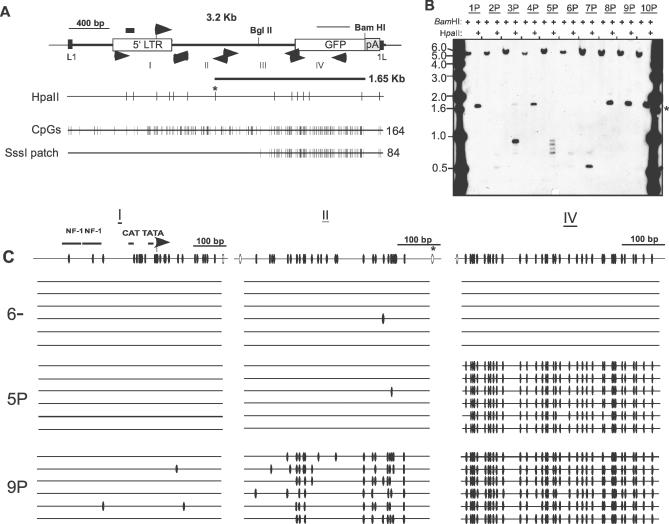
The Low-Expressing Patch-Methylated Clones Show Spreading of Methylation towards the Promoter Region (A) A map of the integrated L1-LTRGFP-1L cassette showing the relevant restriction sites, the probe used for Southern analysis (horizontal bar), the primer pairs used for bisulphite sequencing, and a CpG density map. (B) Southern analysis of genomic DNA isolated from patch-methylated clones digested with BamHI alone or in combination with the methylation-sensitive restriction enzyme HpaII. The double digest reveals bands between ∼1660 bps and ∼530 bps, corresponding to the first HpaII site upstream of the methylated region (shown as an asterisk in [A]), or the fragment size predicted for complete digestion of the cassette, respectively. (C) The methylation status of representative high- (5P) and low- (9P) expressing patch-methylated clones and an unmethylated (6−) control was analyzed in greater detail by bisulphite sequencing, using primers specific for the LTR (I), intron (II), and downstream/methylated regions (IV). The presence of methylation at specific CpG sites is shown (black ovals) for each molecule sequenced (horizontal lines). Empty ovals indicate CpGs embedded in the primer region. The first HpaII site 5′ of the patch-methylated region is marked with an asterisk.

Given the heterogeneity in expression profiles of clones harboring the initially patch-methylated cassette, we next determined the methylation status of the cassettes in each of the clones described in Figure 2. Genomic DNA isolated 35 days post-electroporation was digested with BamHI alone, or in combination with the methylation-sensitive restriction enzyme HpaII, and Southern analysis was conducted using a GFP probe (Figure 3A and 3B). Two classes of clones are readily apparent, one with a relatively high molecular weight band of ∼1.65 kb (clones 1P, 4P, 8P, 9P, and 10P), and another with relatively low molecular weight bands between ∼0.5 and 0.9 kb (clones 2P, 5P, 6P, and 7P). The former is indicative of the absence of methylation at the first HpaII site 5′ of the patch-methylated region, but maintenance of methylation at all HpaII sites 3′ of this site, while the latter is indicative of loss of methylation at some or all of the premethylated sites. Comparison of the methylation state with the expression data, as measured by flow cytometry (see Figure 2B), reveals that clones harboring cassettes with varying degrees of demethylation of the patch-methylated region are all in the high-expressing class, while clones showing maintenance of methylation at the premethylated sites, are all in the low-expressing class of clones.

To characterize the methylation status of representative low- and high-expressing patch-methylated clones in greater detail, we analyzed clones 2P, 5P, 8P, 9P, and the unmethylated control clone 6− by bisulphite sequencing, using primers specific for the LTR (I), GAG (II), “methylation-junction” (III), and GFP/in vitro methylated (IV) regions (Figure 3C; Figure S1; and unpublished data). Sequencing of cloned amplification products revealed that “alleles” of the high-expressing unmethylated control clone 6− and the initially patch-methylated clone 2P are virtually methylation-free, explaining why the latter clone shows an expression profile similar to that of the initially unmethylated cassette. Interestingly, clone 5P, which shows an “intermediate” level of expression, retains the general methylation pattern introduced in vivo, with loss of methylation at some sites in the premethylated region but no spreading upstream of this region.

In contrast, “alleles” of the low-expressing clones 8P and 9P not only retained the initial methylation state at the majority of CpG sites, but also showed significant spreading of methylation upstream of this region (Figure 3C, Figure S1). Thus, the apparently unmethylated HpaII site (as determined by Southern blotting) is embedded within the “de novo*”* methylated domain in these clones. Unfortunately, as this site is within the 3′ primer sequence of the GAG amplicon (see Table S1), the bisulphite data is uninformative for this site. However, several other CpGs in the initially unmethylated promoter proximal region remain unmethylated, despite being flanked by newly methylated sites, indicating that specific CpGs flanking the patch-methylated region are de novo methylated with very different efficiencies.

Surprisingly, in the two low-expressing clones that were analyzed, methylation did not spread beyond ∼320 bps 3′ of the TSS. With the exception of a few dispersed CpGs that are methylated in a subset of sequenced alleles, the promoter and TSS region in these clones remain methylation-free. These data indicate that spreading of methylation to within ∼320 bp of the TSS is sufficient to dramatically reduce transcriptional efficiency from an unmethylated promoter, while methylation ∼1,000 bp 3′ of the TSS has a relatively modest effect on transcription.

Previously, we found that the histone deacetylase (HDAC) inhibitor Trichostatin A (TSA) dramatically increased transcription from MoMuLV proviral clones constitutively expressing at a low level, but is incapable of activating expression in the same clones at a later time point when DNA methylation has accumulated in the TSS region [9]. These results indicate that deacetylation of histones enhances transcription from active promoters, but is ineffective at promoting transcription from promoters that are methylated. Treatment of representative clones (3− and 6−) harboring the unmethylated L1-LTRGFP-1L cassette with 50nmol TSA for 48 h yielded a dramatic increase in expression level (Figure S2). In contrast, the same treatment of the low-expressing clones 8P and 9P yielded no increase in GFP expression, indicating that in the presence of promoter-proximal methylation, inhibition of HDAC activity is not sufficient for transcriptional induction.

**Figure 4:**
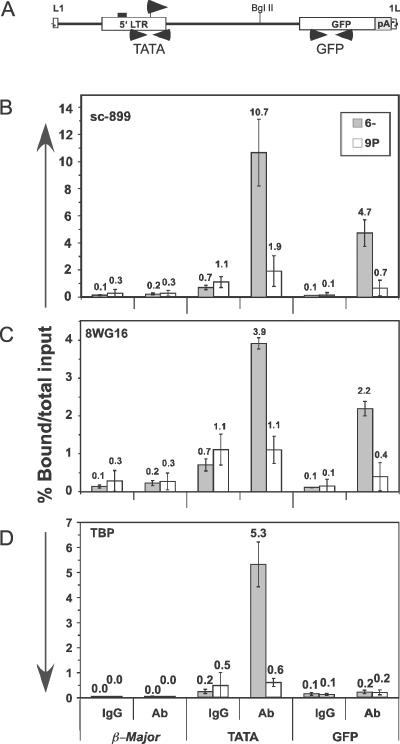
Promoter-Proximal Methylation Affects RNAPII Recruitment and TBP Binding Chromatin isolated from unmethylated and patch-methylated clones 6− and 9P, respectively, was immunoprecipitated using antibodies (Abs) specific for the N-terminal domain of RNAPII (sc-899), the unphosphorylated CTD of RNAPII (8WG16), TBP, or control rabbit IgG. Quantitative real-time PCR was conducted using the transgene specific primers shown (A), or a control primer pair specific for the endogenous *β-maj* gene. The mean percent (+/− standard deviation) of bound material/total input chromatin for two to three independent chromatin preparations is shown for each ChIP experiment. No enrichment of RNAPII was detected in the silent *β-maj* gene in either clone with sc-899 (B) or 8WG16 (C), demonstrating the low level of background detected under the conditions used. Analysis of the TATA box and GFP regions of the transgene reveals significant enrichment of RNAPII exclusively in the unmethylated, highly transcribed clone, particularly at the 5′ end of the transgene. Similarly, TBP binding is detected exclusively in the promoter region of the unmethylated clone (D), suggesting that inhibition of transcription of the patch-methylated cassette occurs as the level of transcription initiation.

To determine whether methylation in close proximity to the promoter region influences recruitment of RNAPII to the transgene, formaldehyde cross-linked chromatin was generated from a representative unmethylated control clone (6−) and a low-expressing patch-methylated clone (9P). Chromatin immunoprecipitation (ChIP) was performed using antibodies specific for the N-terminal domain (sc−899) or the unphosphorylated C-terminal domain (CTD) (8WG16) of RNAPII. Quantitative real-time PCR was conducted using primers specific for the transgene (Figure 4A), as well as for the endogenous *β-major (β-maj) globin* gene, which is not expressed in uninduced MEL cells [21]. Analysis of the data generated from two independent chromatin preparations reveals that, as expected, no enrichment of RNAPII is detected in the promoter region of the *β-maj* gene, relative to control immunoglobulin G (IgG) (Figure 4B and 4C). While clone 9P also shows no enrichment of RNAPII in the promoter (TATA) and downstream (GFP) regions of the transgene, the unmethylated clone shows significant enrichment in both regions using both α-RNAPII reagents (Figure 4B and 4C). Not surprisingly, a higher level of enrichment of RNAPII was detected in the promoter than in the downstream region. Similar results were obtained using an antibody specific for RNAPII phosphorylated on Serine 5 of the CTD, the elongation-competent form of the holoenzyme (Figure S3). Taken together, these results reveal that methylation beginning ∼320 bp downstream of the TSS is sufficient to inhibit recruitment of RNAPII to the unmethylated transgene promoter.

To determine whether the failure to recruit RNAPII is the result of the inhibition of formation of the preinitiation complex (PIC), we next carried out ChIP analysis of TBP, a subunit of the transcription initiation factor (TFIID) complex that binds to the TATA box and initiates formation of the PIC. Analysis of the data generated from two independent chromatin preparations reveals that relative to control IgG, no TBP is detected in the promoter region of the endogenous *β-maj* gene or in the GFP region of the transgene in either clone (Figure 4D). However, while the unmethylated clone shows significant enrichment of TBP in the TATA box region, clone 9P shows no enrichment. These data indicate that the reduced expression observed in the patch-methylated clone is the result of transcriptional inhibition at a step prior to recruitment of TFIID to the promoter region.

Given that recruitment of RNAPII and TFIID to the promoter region of the patch-methylated transgene is clearly inhibited, we next investigated whether the histone marks normally associated with “active” genes are similarly affected. The 5′ ends of actively transcribing genes are marked by histone H3 trimethylated (H3K4me3) and dimethylated (H3K4me2) on K4 [22], and several of the HMTases with specificity for H3K4 are associated with the elongating RNAPII holoenzyme. Interestingly, methylation of H3K4 precedes acetylation of H3 in the promoter regions of inducible genes [23], and a low but clearly detectable level of H3K4me3 is found associated with the 5′ end of some genes “poised” for transcription [22,24]. To determine whether promoter-proximal DNA methylation influences H3K4 methylation state, two independent ChIP experiments were conducted using antibodies specific for H3K4me3 or H3K4me2 (Figure S3) and primers specific for the transgene or the endogenous pancreatic *Amylase 2 (Amy2)* gene, which is transcriptionally silent in MEL cells. In the intragenic regions analyzed, H3K4me3 enrichment is significantly higher in the transcriptionally active unmethylated control, likely the result of transcription-coupled deposition of H3K4me3. Surprisingly, enrichment of this mark in the promoter region is 2-fold higher in the patch-methylated than the unmethylated clone, perhaps as a consequence of decreased turnover of nucleosomes in the promoter region of the patch-methylated cassette. Analysis of H3K4me2 revealed low but significant levels of enrichment on both clones, with clone 9P showing a ∼2–4-fold lower level of enrichment throughout the transgene. These results reveal that while DNA methylation starting ∼320 bp 3′ of the TSS yields a lower level of H3K4 methylation in the transcribed region of the cassette, this mark is still present in the promoter region.

We next tested whether acetylation of the H3 tail, another mark associated with “active” chromatin, is affected by promoter-proximal methylation. Acetylation of histone tails neutralizes the positive charge on lysines and is believed to promote an allosteric change in nucleosome conformation that renders nucleosomal DNA more accessible to the transcription machinery [25,26], perhaps by altering higher order chromatin structure [27]. While binding of TBP to the TATA box is severely inhibited by incorporation of template DNA into a nucleosome [28], histone acetylation can facilitate TBP binding to a naturally positioned nucleosome [29]. Furthermore, in vitro and in vivo experiments are consistent with the model that histone acetylation is an early step in the cascade of events leading to transcription initiation [30–33]. We hypothesized that a condensed chromatin structure extending 5′ of the methylated region and/or a methylation-mediated alteration in nucleosome positioning may be responsible for the observed effect on TBP binding and transcription initiation.

**Figure 5:**
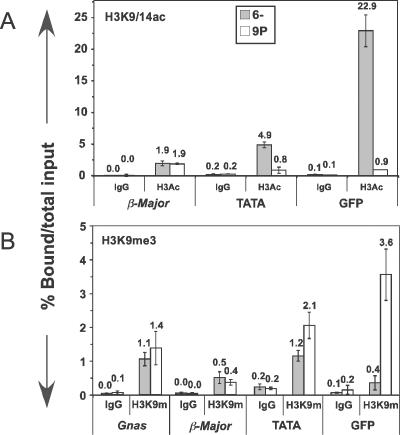
Promoter-Proximal DNA Methylation Leads to Hypoacetylation of H3K9/14 across the Transgene and Local H3K9 Trimethylation Chromatin was generated from clones 6− and 9P, and ChIP was conducted using antibodies specific for H3 acetylated on K9/K14, H3 trimethylated on K9 or control rabbit IgG. Quantitative real-time PCR was carried out on the immunoprecipitated DNA using primers specific for the endogenous *Gnas* and/or *β-maj* genes as internal controls and the TATA box or GFP regions of the transgene (shown in Figure 4A). For each antibody, values shown represent the mean percent (+/− standard deviation) of bound material/total input chromatin for three independent experiments. (A) Analysis of the TATA box and GFP regions of the transgene reveals enrichment of H3K9/14Ac (H3Ac) exclusively in the highly transcribed unmethylated cassette. The level of enrichment of H3K9/14Ac in the *β-maj* gene promoter region was similar between the two clones, indicating that the immunoprecipitations worked with similar efficiencies. (B) Analysis of the TATA box and GFP regions of the transgene reveals a significantly higher level of H3K9me3 (H3K9m) enrichment exclusively in the GFP region of clone 9P. As this region shows a high density of DNA methylation, these results reveal that DNA methylation is sufficient to promote H3K9 trimethylation in this euchromatic region. The levels of enrichment of H3K9me3 at *Gnas* and *β-maj* was similar between the two clones, indicating that the immunoprecipitations worked with similar efficiencies.

We isolated chromatin from clones 6− and 9P and determined the acetylation state of H3 on K9 and K14 (H3K9/K14ac), the latter of which is the major substrate of the mammalian p300, PCAF and GCN5 histone acetyltransferases [34,35]. Similar levels of enrichment of H3K9/K14ac were detected at the endogenous *β-maj* gene in both the unmethylated and patch-methylated clones (Figure 5A), indicating that the efficiency of the immunoprecipitation was similar for both clones. Surprisingly, analysis of the TATA and GFP regions of the transgene revealed significant enrichment of H3 acetylation exclusively in the unmethylated clone, despite the fact that the promoter/TATA box region of the patch-methylated cassette is also unmethylated.

Given the absence of H3K9/K14ac throughout the patch-methylated cassette, and the reported association of DNA methylation and H3K9 methylation [36–38], we next tested whether the patch-methylated cassette is marked by H3K9me3, using the endogenous *Gnas* gene, previously shown to be marked by H3K9me3 in MEL cells [39], as a positive control (Figure 5B**)**. Analysis of the *Gnas* gene revealed similar levels of enrichment in the unmethylated and patch-methylated clones (∼26- and 23-fold, respectively, relative to the IgG control), indicating that the H3K9me3 ChIP worked with similar efficiency in both chromatin preparations. A low level of enrichment, was also detected in the promoter region of the endogenous *β-maj* gene in both clones, consistent with the previous report showing H3K9me3 1 kb 3′ of the promoter region in uninduced MEL cells [40]. Analysis of the TATA box region of the transgene reveals that both clones 6− and 9P show a modest level of enrichment for H3K9me3. In contrast, analysis of the GFP region reveals a high level of H3K9me3 enrichment (∼22-fold relative to IgG) exclusively in clone 9P. The unmethylated clone shows a relatively low level of enrichment in the same region, ∼4.7-fold relative to IgG. Given the size of chromatin fragments generated via sonication, the slightly higher level of enrichment detected in the TATA region of clone 9P may be the result of amplification of template fragments bearing H3K9me3 in the downstream DNA methylated region. These data reveal that targeting of H3K9me3 to “euchromatic” regions in mammalian cells may be facilitated by the presence of DNA methylation per se, and that methylation-associated deposition of this mark may not spread beyond the DNA methylated regions.

**Figure 6:**
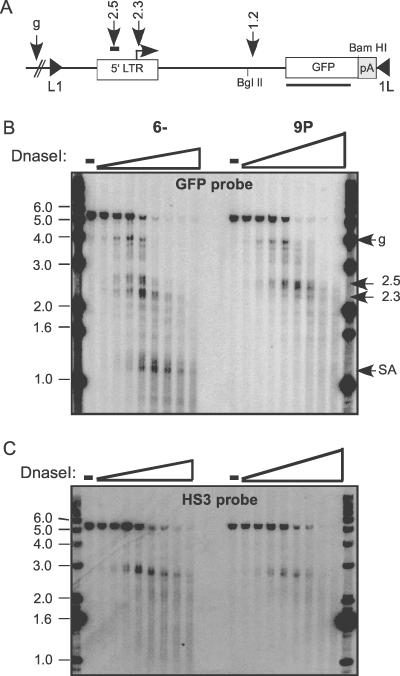
Promoter-Proximal DNA Methylation Influences the Chromatin Structure of the Unmethylated Promoter Region To compare the chromatin structure of the unmethylated versus patch-methylated transgenes, intact nuclei from clones 6− and 9P respectively, were digested with increasing concentrations of DNase I. Subsequently, genomic DNA was isolated and digested with BamHI. Digested DNA was resolved on a 0.8% agarose gel. A map of the transgene, GFP probe, and HS sites is shown (A). The location of transgene-specific DNase HS sites was determined via Southern hybridization using a GFP probe (B). Predicted HS sites at the proviral enhancer and promoter, 2.5 kb and 2.3 kb upstream of the BamHI site, respectively, are shown. Note that the promoter-specific HS site is absent in clone 9P, even in the presence of relatively high concentrations of DNase I. Two additional HS sites, one site that is common to both clones and maps to a genomic (g) site (within a Charlie4 DNA transposon) ∼3 kb upstream of the enhancer HS site, and a second that is unique to the 6− clone and maps to the splice acceptor (SA) site within the transgene (∼1.2 kb upstream of the BamHI site) were also detected. To demonstrate comparable efficiencies of DNase I digestion in the two clones, the blot shown in (B) was stripped and reprobed with a probe specific for the “HS3” hypersensitive site in the endogenous β-globin Locus Control Region (C).

To directly determine whether the observed inhibition of transcription in the low-expressing patch-methylated clones is associated with an altered chromatin structure in the upstream unmethylated CpG island promoter/TSS region, DNase I hypersensitivity (HS) analysis was conducted. Previous experiments have shown that a transcriptionally active MoMuLV LTR harbors two HS sites, one in the upstream enhancer region, and another around the TSS [41,42]. Nuclei from clones 6− and 9P were isolated and treated with DNase I. Subsequently, genomic DNA was extracted, digested with BamHI, and analyzed by Southern blot using the indirect end-labeling technique (Figure 6A).

The unmethylated clone showed a predominant band at 2.3 kb, and an additional band at 2.5 kb, corresponding to the TSS and the enhancer regions, respectively. In contrast, the clone bearing promoter-proximal DNA methylation showed a band of comparable intensity in the enhancer region, but only a faint HS site around the TSS, indicating that the chromatin structure around the TSS is indeed altered by DNA methylation ∼320 bp downstream of this site (Figure 6B). To establish that the difference in DNase I digestion patterns was not the result of a difference in the efficiency of digestion, the blot shown in 6B was subsequently stripped and reprobed with a probe specific for the endogenous “HS3” site in the Locus Control Region of the β-globin locus (Figure 6C). A single DNase I-dependent band is present in clone 6− and 9P samples, demonstrating that both clones were digested with comparable efficiency.

**Figure 7:**
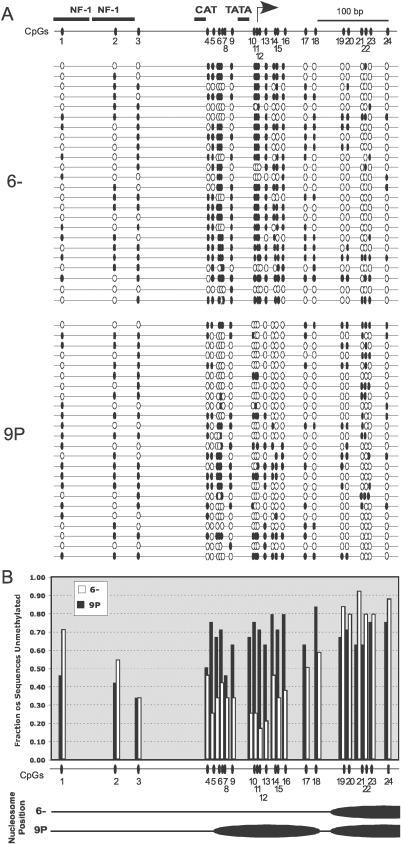
Promoter-Proximal DNA Methylation Influences Nucleosome Positioning in the Unmethylated Promoter Region To determine nucleosome positioning around the promoter region, nuclei from clones 6− and 9P were treated in parallel with M.SssI for 15 min. Subsequently, genomic DNA was isolated and analyzed by bisulphite sequencing using primers flanking the promoter/TSS region. For each clone, 24 molecules were cloned and sequenced. Methylated and unmethylated CpGs are depicted as filled and open ovals, respectively (A). The fraction of molecules harboring an unmethylated cytosine at each CpG in the sequenced region is plotted in (B). Note the dramatic difference in methylation efficiency spanning the region from ∼CpG #5 to ∼CpG #18 (a distance of 148 bps), particularly those sites flanking the TSS at ∼CpG #11. Based on these data, a diagram showing the predicted predominant sites of nucleosome occupancy (ovals) for each clone is shown. The enhancer region (CpGs #1–3) is likely to be constitutively nucleosome-free, consistent with the presence of a DNase HS site in this region in both clones.

To determine whether the loss of the TSS-specific HS site reflects an alteration in nucleosome positioning at the TSS of the patch-methylated cassette, “methylase-based single promoter analysis” (M-SPA), a method recently developed by Fatemi et al*.* [43], was conducted using nuclei isolated from clones 6− and 9P. M-SPA involves treatment of isolated nuclei with the cytosine-C5 CpG-specific DNA methyltransferase SssI (M.SssI), followed by genomic bisulphite sequencing of individual progeny DNA molecules. In the context of chromatin, the accessibility of individual CpGs to M.SssI activity is dependent upon nucleosome positioning or the presence of bound transcription factors [44]. Patches of ∼150 bp of relatively under-methylated regions correlate with the approximate positions of nucleosomes on individual sequenced molecules [43]. As the promoter region of the transgene is virtually methylation-free in both the patch-methylated and unmethylated clones (see Figure 3), the methylation detected predominantly reflects the accessibility of individual CpGs to M.SssI activity. Bisulphite analysis of isolated genomic DNA treated with M.SssI for 5 min revealed complete methylation of all 24 CpGs in the promoter region (unpublished data). In contrast, analysis of isolated nuclei treated with M.SssI for 15 min revealed a clone-dependent methylation pattern (Figure 7A). While clone 6− shows a relatively low level of methylation downstream of the TSS, clone 9P showed a relatively low level of methylation across a ∼150-bp region centered on the TSS (Figure 7B). These distinct patterns of methylation sensitivity likely reflect the positioning of a nucleosome over the promoter/TSS region in clone 9P, and the absence of a nucleosome in this region in the unmethylated, highly transcribed control. Taken together, these data reveal that the chromatin structure around the unmethylated TSS is indeed altered in cells harboring DNA methylation ∼320 bp 3′ of this region, demonstrating that this epigenetic mark can act at a distance to disrupt nucleosome positioning and in turn, transcription initiation in mammalian cells.

## Discussion

Here we utilized RMCE to target a patch-methylated construct into a defined chromosomal region. Spreading of methylation towards the TSS in a subset of clones allowed us to study the influence of methylation on transcription when present at different distances downstream of a methylation-free promoter. When methylation spreads to within ∼320 bp of the TSS, expression is dramatically reduced, relative to an unmethylated cassette integrated at the same site. Clones bearing such promoter-proximal methylation show loss of RNAPII and TBP binding and a reduction in the level of H3K4me3 in the transcribed region. Surprisingly, while H3K9me3 is enriched in the patch-methylated region, this mark does not spread upstream into the unmethylated promoter, despite the fact that this region is hypo-acetylated on H3K9/14. Nevertheless, the observed loss of a promoter-specific DNase I HS site and alteration of nucleosome positioning around the promoter/TSS region reveals that DNA methylation beginning 320 bp—three nucleosomes—downstream of the TSS generates a chromatin structure in the methylation-free promoter region that precludes efficient transcription initiation.

Previously, we conducted a similar set of experiments using a construct with the p16 promoter driving expression of the GFP gene [19]. This construct, which differs from the construct used in this study at the promoter region only, was patch-methylated in vitro, in the same region as the L1-LTRGFP-1L construct and integrated at the same genomic site via RMCE. In contrast to the LTR-based construct, only one GFP+ population was detected by flow cytometry. Analysis of the methylation status of GFP+ clones harboring the p16 promoter-based construct, which showed a decrease in expression of ∼40% relative to unmethylated control clones, revealed no spreading of methylation upstream of the premethylated region ∼1 kb downstream of the promoter, and no apparent effect on transcription initiation rate, as determined by polII ChIP and run-on analyses of the transcribed region upstream of the patch-methylated domain. Given that the level of expression from the unmethylated LTR is approximately five times greater than that of the unmethylated p16 promoter (at the RL5 integration site), it is quite possible that methylation spreading inhibited transcription from the p16 promoter to such an extent that the level of GFP expression was below the threshold for detection by flow cytometry. If so, this class of clones was inadvertently excluded from our original study, as only cells showing detectable GFP expression were chosen for further analysis.

**Figure 8:**
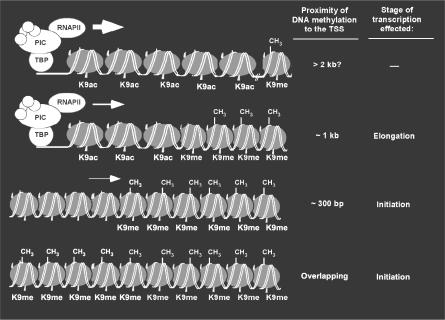
Model of the Relationship between Promoter-Proximal DNA Methylation and Transcriptional Efficiency Four alternative DNA methylation states are shown, along with the acetylation (K9ac) and methylation (K9me) states of H3K9. In the presence of “distal” DNA methylation (>1–2 kb from the TSS), transcription may not be effected. This would allow for methylation and silencing of intronic parasitic elements, such as SINE and LINES or “cryptic promoters”, while allowing for efficient transcription of bona fide genes. Dense DNA methylation on the order of 1 kb from the TSS may have a modest effect on elongation efficiency, perhaps by altering the chromatin structure of the promoter proximal region, as we have shown previously [19]. Promoter proximal methylation (∼300 bp from the TSS) may have a dramatic effect on transcription initiation, as a result of altering promoter chromatin structure, as described here. Methylation of the promoter region itself leads to robust silencing of the gene, as is frequently observed for CpG island promoters in cancer cells.

In a subpopulation of cells harboring the L1-LTRGFP-1L cassette described here, a region upstream of the in vitro methylated domain was de novo methylated, yielding a dramatic decrease in expression that was nevertheless above the threshold for detection by flow cytometry. Why methylation spreading did not extend into the promoter region remains to be determined. Given the persistence of H3K4 methylation in the promoter region of the patch-methylated cassette, it is tempting to speculate that this “active” mark may serve in part to protect this region from de novo DNA methylation. Regardless, these data reveal that while transcription is only modestly reduced in the presence of a dense patch of methylation ∼1 kb downstream of the TSS, methylation within ∼320 bp of the TSS is sufficient to dramatically reduce transcription initiation efficiency, results consistent with those of Graessmann and colleagues, who found using microinjection of premethylated HSV-based reporter constructs that methylation of specific CpGs >570 bp downstream of the TSS had no effect on expression, while methylation of CpGs within 570 bp of the TSS had a dramatic effect on transcription [15]. Similarly, using a transiently transfected luciferase reporter, Hisano et al*.* showed that methylation of a region ∼100 bp downstream of the TSS of the minimal promoter (which lacks CpGs altogether) of the murine *Tact1* gene yielded a dramatic reduction in expression, relative to an unmethylated control [45]. Taken together, these observations indicate that methylation of the promoter region per se is not a prerequisite for DNA methylation-mediated transcriptional inhibition. As outlined in the model described in Figure 8, we propose that the distance of DNA methylation downstream of the TSS dictates the nature and level of transcriptional inhibition, with a modest effect on elongation efficiency occurring in the presence of dense DNA methylation on the order of 1 kb downstream of the TSS [19], and a dramatic decrease in initiation efficiency occurring in the presence of DNA methylation on the order of 300 bp downstream of the TSS.

Recruitment of HDAC and/or H3K9 MTase complexes to the methylated region may play a role in inhibiting transcriptional initiation at a distance. However, while we did detect a high level of enrichment of H3K9me3 in the DNA methylated/GFP region in the patch-methylated clone analyzed here, enrichment of this mark was only 2-fold higher in the promoter region of the patch-methylated cassette than the control unmethylated cassette, indicating that H3K9me3 acts at a distance to prevent recruitment of TBP, or is not responsible for the observed initiation block. As Vakoc et al*.* recently showed that H3K9me3 is frequently found in the transcribed regions of actively transcribing genes [40], we favor the latter possibility. Regardless, given that the unmethylated control cassette shows a relatively low level of enrichment of the H3K9me3 mark in the otherwise identical but unmethylated GFP region, our experiments clearly reveal that the presence of a short patch of DNA methylation is sufficient to recruit an HMTase with H3K9me3 activity to a “euchromatic” region in mammalian cells. Consistent with the hypothesis that DNA methylation can act “upstream” of H3K9 trimethylation in mammalian cells, Feng and colleagues recently showed that the presence of CpG dinucleotides is required for maintenance of H3K9 trimethylation [39].

Genome-wide analysis of the distribution of H3K9/K14 acetylation reveals that this mark typically extends ∼1 kb downstream of TSSs, and along the entire length of CpG islands [46]. Acetylation of histone tails in promoter regions facilitates efficient remodeling of nucleosomes via recruitment of SWI/SNF chromatin remodeling complexes and/or TFIID [29,33,47,48], and a recent analysis of PIC formation in 29 ENCODE regions in human cells reveals a very strong correlation between H3 acetylation and the presence of a PIC [49]. As the unmethylated promoter region of the patch-methylated cassette described here is hypo-acetylated relative to the identical but unmethylated control cassette integrated at the same site, we propose that the observed decrease in histone H3 acetylation reduces nucleosome “fluidity” in the promoter proximal region, which inhibits repositioning of the nucleosome around the TATA-box and in turn, recruitment of TBP.

Support for this hypothesis comes from the DNase I and M-SPA analyses described here. For a number of genes, formation of promoter DNase I HS sites precedes active transcription [50–52], perhaps reflecting the activity of chromatin remodeling complexes. Indeed, maintenance of expression from the MoMuLV LTR requires Bramha, the catalytic subunit of one of the mammalian SWI/SNF complexes [53]. Clones bearing methylated CpGs ∼300bp downstream of the TSS showed loss of the HS site normally found at the TSS and the presence of a nucleosome centered on the TSS, clearly demonstrating that promoter-proximal methylation can alter the chromatin structure of an unmethylated upstream promoter.

Recently, Frigola and colleagues demonstrated that silencing of all of the genes within a 4-Mb band of Chromosome 2q.14.2 occurs frequently in colorectal cancer [54]. Surprisingly, while several “CpG island suburbs” within this region harbor genes with hypermethylated CpG island promoters, other genes within this band are silenced despite the absence of promoter-specific DNA methylation, indicating that DNA methylation within promoter regions per se is neither a prerequisite nor a necessary consequence of transcriptional silencing in cancer. In light of the results reported here, it would be of interest to analyze the methylation state of the regions flanking the unmethylated but silenced CpG island promoters in this region.

Bird and colleagues noted some time ago that a high density of CpGs extend 3′ of the TSSs associated with CpG islands [55]. Indeed, recent bioinformatic analyses reveals that a relatively high density of CpGs is frequently found to extend on the order of 1 kb downstream of the TSSs of mammalian promoters [6,8], indicative of the absence of methylation within these regions, at least in the germ line [56]. Our results reveal that a methylation-free region extending downstream of the TSS may be necessary for efficient promoter activity, perhaps explaining why CpG islands promoters are structured in this way.

## Materials and Methods

### Generation and in vitro methylation of the L1-LTRGFPpolyA-1L cassette.

A HindIII to BamHI fragment including the MoMuLV LTR and GFP from the previously described L1-MFGGFP-1L plasmid [42] was cloned into the vector L1polylinker1L. Subsequently, a fragment containing the SV40 polyA signal was inserted into the BamHI site at the 3′ end of the GFP gene of this intermediate construct, yielding the L1-LTRGFP-1L construct used in this study. To generate the “patch”-methylated cassette, this vector was digested with BglII and BamHI, yielding two fragments, one containing the LTR and transcribed GAG sequence, and the other the GFP gene and plasmid backbone. The latter fragment was methylated in vitro with M.SssI and ligated to the unmethylated promoter fragment, generating a plasmid in which the TSS of the LTR is ∼1.0 kb from the 5′ end of the methylated region. To confirm that the methylation reaction was carried to completion, methylated DNA was, digested with the methylation-sensitive enzyme HpaII following organic extraction and visualized by electrophoresis on an agarose gel (unpublished data).

### Tissue culture and RMCE.

Unmethylated and patch-methylated plasmid was introduced into RL5 MEL cells [17] by electroporation, and selected in the presence of ganciclovir as previously described [11]. GFP+ cells were isolated by FACS, cloned by limiting dilution, and screened for successful Cre-mediated exchange by Southern blotting. For treatment with TSA (Wako Pure Chemicals Industries, http://www.wako-chem.co.jp/english), aliquots dissolved at 5 mg/ml in methanol and stored at −20 °C were thawed, diluted in fresh complete medium, and added to MEL cells at a final concentration of 50 nmol. Using this protocol, more than 90% of cells are viable, as measured by propidium iodide (PI) staining.

### Flow cytometry and RT-PCR.

For FACS analyses, MEL cells were processed as previously described [42]. Data on at least 10,000 viable cells (as determined by PI staining) were collected for each sample and analyzed using FlowJo software (Treestar, http://www.treestar.com). For RT-PCR, total RNA was isolated from 5 × 10^6^ cells using the RNeasy kit (Qiagen, http://www.qiagen.com) following the manufacturer's protocol. Quantitative RT-PCR was carried out using random hexamer as described [42]. For second strand synthesis, each 25 μl reaction was supplemented with 1 μCi of [α-^32^P]dCTP (NEN). Primers spanning the transgene intron (see Table S1 for primer sequences) were used in a duplex PCR reaction with primers specific for the endogenous *β-actin* gene as an internal control. Amplification products were quantified via storage phosphor imaging using a Typhoon 8600 and ImageQuant software (Molecular Dynamics, http://www.ump.com/mdynamic.html).

### Southern blotting and bisulphite analysis.

Preparation of genomic DNA, the DNA probe, restriction digests, and membrane transfers were performed as described previously [42]. Clones harboring a single copy integrant at the RL5 integration site were identified by digestion of genomic DNA (isolated at day 21 post-RMCE) with BamHI, which cuts once in the MFGGFP provirus, followed by Southern blot analysis using the indirect end-labeling technique with the GFP probe. Methylation status of the cassette was determined by digestion of genomic DNA with BamHI alone or in combination with the methylation sensitive enzyme HpaII. For bisulphite analyses, genomic DNA was digested with BamHI, purified by phenol/chloroform extraction, denatured, treated with bisulphite as described previously [9], and subject to nested or semi-nested PCR using primers designed to amplify bisulfite-converted template. Primers used for the LTR (I), GAG (II), and GFP regions (IV) are listed in Table S1. PCR products were cloned via T/A cloning using the pGEM-T easy kit (Promega, http://www.promega.com), and individual inserts were sequenced using the Big Dye version 3.1. Sequencing data was analyzed using Sequencher software (Gene Codes, http://www.genecodes.com).

### DNase I HS analysis.

DNase I digestion of nuclei was performed as described previously [57], using from 0.67–7.67 μg DNase I/ml. DNase I digested genomic DNA was purified and digested with BamHI, prior to electrophoresis on a 0.8% agarose gel. The GFP probe used for hybridization was generated by digestion of the plasmidL1-MFG-*GFP-*1L with NcoI and BamHI, yielding a restriction fragment including the complete 720-bp *GFP* gene. The blot was stripped by pouring a boiling solution of 5% SDS and 5 mmol NaOH over the blot and allowing it to cool to room temperature. After repeating this step with 0.5% SDS only, the blot was rinsed with 2× SSC. The blot was subsequently exposed to film to establish the efficiency of stripping. The probe used for rehybridization was generated by digestion of the plasmid BSKSIImHS3TIM with EcoRI and BamHI, generating a 360-bp fragment that is specific for the “HS3” region of the murine ß-globin Locus Control Region.

###  M-SPA.

M-SPA was conducted as described previously [43], with minor modifications. For in vitro methylation of purified DNA, 6 μg of EcoRI-digested genomic DNA was treated with 60 Units M.SssI for 5 min and the reaction stopped with 2× lysis buffer. For M.SssI treatment of nuclei, 1 × 10^7^ cells were harvested, and nuclei were isolated via dounce homogenization. Intact nuclei were treated with 60 Units M.SssI for 15 min, and processed as described [43]. Subsequently, 500 ng of genomic DNA was subject to bisulphite conversion using the EZ-DNA methylation Gold kit (Zymo Research, http://www.zymoresearch.com) and processed as described above.

### ChIP analyses.

To generate cross-linked chromatin, 2.4–4 × 10^7^ exponentially growing cells were incubated in the presence of 1% (v/v) formaldehyde for 10 min at 37 °C. Chromatin was generated and ChIP conducted as described previously [58]. Polyclonal antibodies specific for TBP (kindly provided by N. Hernandez) H3K9/14ac (5 μg; Upstate, 06–599), which is reported to preferentially recognize H3 acetylated on K9 [59], H3K4me2 (5 μl) (Upstate, 07–030, http://www.upstate.com), H3K4me3 (4.5 μg) (Abcam, ab8580, http://www.abcam.com), and H3K9me3 (4.5 μg) (Upstate, 07–442) were used in combination with control, purified rabbit IgG (20 μg) (Sigma, http://www.sigmaaldrich.com).

To generate cross-linked chromatin for ChIP with antibodies recognizing RNAPII, 1.6 × 10^7^ exponentially growing cells were incubated in the presence of 1% (v/v) formaldehyde for 10 min at 25 °C, and ChIP was conducted, as described previously [60]. Sonicated fragments were generated using a Bioruptor water bath sonicator (Diagenode, http://www.diagenode.com), with chromatin fragments ranging in size from 250–750 bp, as determined by electrophoresis through a 0.8% agarose-Tris-borate-EDTA gel and ethidium bromide staining. α-RNAPII antibodies used include: the polyclonals SC-899 (2 μg) (Santa Cruz, http://www.scbt.com) and 8WG16 (∼25 μg) (Covance, MMS-126R, http://www.covance.com), specific for the amino- and carboxy-termini, respectively, of the large subunit of RNAPII and the monoclonal H14 (20 μg; Covance, MMS-134R), specific for RNAPII phosphorylated on S5 of the CTD.

For experiments using the α-RNAPII antibodies SC-899 and 8WG16 as well as, the TBP, H3K9/14ac and H3K9me3 specific antibodies, enrichment via ChIP was determined by real-time quantitative PCR using an Opticon 2 thermal cycler (Bio-Rad, http://www.bio-rad.com), with EvaGreen (Biotium, http://www.biotium.com), and hot-start Taq polymerase (Fermentas, http://www.fermentas.com). Primers specific for the TATA box and GFP regions of the transgene were used, as well as primers specific for the endogenous *Gnas* and *β-maj* genes (sequences shown in Table S1). Conditions are available upon request. The percentage of material bound, relative to total input, was determined using the standard curve method.

For ChIP using the H14, H3K4me2, and H3K4me3 antibodies, quantitative duplex PCR was performed using a PerkinElmer 9700 thermocycler (http://www.perkinelmer.com), as described [42]. Conditions of linear amplification were determined empirically for all primer combinations. Each 25 μl reaction was supplemented with 1 μCi of [α-^32^P]dCTP (PerkinElmer). Primers specific for the LTR, GAG, and GFP regions of the transgene, as well as the endogenous *Amy2* gene are shown in Table S1. The reaction product was subject to electrophoresis on a 5% (v/v) nondenaturing polyacrylamide gel, and the amount of amplified product was quantified as described for RT-PCR. To determine “fold-enrichment” values for a given region in the cassette, the ratio of the two PCR products (transgene/control) was calculated for the antibody bound fraction and normalized to the ratio obtained from the input material. Relative enrichment values were subsequently calculated by taking the ratio of the fold-enrichment values of the unmethylated/methylated samples in each of the regions analyzed.

## Supporting Information

Figure S1 Methylation State of Two Additional Patch-Methylated Clones A map of the integrated L1-LTRGFP-1L cassette showing the primer pairs used for bisulphite sequencing is shown. The methylation status of regions I, II, and III of an initially patch-methylated clone that has undergone demethylation in vivo (2P) and an additional low-expressing clone (8P) was determined by bisulphite sequencing, as described in Figure 1.
(161 KB PDF)

Figure S2 Promoter-Proximal DNA Methylation Abolishes TSA Inducibility of the Transgene To determine whether the transcriptional inhibition associated with DNA methylation exclusive of the promoter could be reversed by inhibiting HDAC activity, two patch-methylated clones (8P and 9P) and two control unmethylated clones (6−, 3−) were treated with 50 nmol TSA. Cells were analyzed for GFP expression 48 h post-induction by flow cytometry and median GFP fluorescence values are shown (ND, not done). In contrast to the unmethylated controls, TSA does not induce expression of the low-expressing patch-methylated clones.
(35 KB PDF)

Figure S3 Promoter-Proximal Methylation Affects RNAPII Recruitment and H3K4 Methylation (A) A map of the integrated L1-LTRGFP-1L transgene, including the primer pairs used for ChIP is shown. Chromatin isolated from clones 6− and 9P was sonicated and subject to ChIP analyses using antibodies specific for RNAPII phosphorylated at S5 of the CTD (polII-S5) (B), or histone H3 di- (H3K4me2) (C) or tri- (H3K4me3) (D) methylated on K4. Fold-enrichment values (+/− standard deviation) relative to the transcriptionally inert *Amy*2 gene were determined as described in the materials and methods section for two independent experiments.
(55 KB PDF)

Table S1 Primers Used in This Study (42 KB PDF)

### Accession Numbers

The GenBank (http://www.ncbi.nlm.nih.gov/gquery/gquery.fcgi) accession numbers for the genes and genomes discussed in this paper are MoMuLV (AF033811); *β-major (β-maj) globin* (NM_008220); *Gnas* (NM_010309); and *Amy2* (NM_009669).

## Abbreviations

ChIP: chromatin immunoprecipitation
CTD: C-terminal domain
GFP: green fluorescent protein
H3K4me2: histone H3dimethylated
H3K4me3: histone H3 trimethylated
HDAC: histone deacetylase
HS: hypersensitivity
IgG: Immunoglobulin G
LTR: long terminal repeat
MEL: murine erythroleukemia
MoMuLV: Moloney Murine Leukemia Virus
M-SPA: methylase-based single promoter analysis
M.SssI: methyltransferase SssI
PIC: preinitiation complex
RMCE: recombinase-mediated cassette exchange
RNAPII: RNA polymerase II
TBP: TATA-box-binding protein
TFIID: transcription initiation factor IID
TSA: Trichostatin A
TSS: transcription start site

## References

[pgen-0030027-b001] Li E, Bestor TH, Jaenisch R (1992). Targeted mutation of the DNA methyltransferase gene results in embryonic lethality. Cell.

[pgen-0030027-b002] Okano M, Bell DW, Haber DA, Li E (1999). DNA methyltransferases Dnmt3a and Dnmt3b are essential for de novo methylation and mammalian development. Cell.

[pgen-0030027-b003] Goll MG, Bestor TH (2005). Eukaryotic cytosine methyltransferases. Annu Rev Biochem.

[pgen-0030027-b004] Bird A (2002). DNA methylation patterns and epigenetic memory. Genes Dev.

[pgen-0030027-b005] Takai D, Jones PA (2002). Comprehensive analysis of CpG islands in human chromosomes 21 and 22. Proc Natl Acad Sci U S A.

[pgen-0030027-b006] Saxonov S, Berg P, Brutlag DL (2006). A genome-wide analysis of CpG dinucleotides in the human genome distinguishes two distinct classes of promoters. Proc Natl Acad Sci U S A.

[pgen-0030027-b007] Rollins RA, Haghighi F, Edwards JR, Das R, Zhang MQ (2006). Large-scale structure of genomic methylation patterns. Genome Res.

[pgen-0030027-b008] Aerts S, Thijs G, Dabrowski M, Moreau Y, De Moor B (2004). Comprehensive analysis of the base composition around the transcription start site in Metazoa. BMC Genomics.

[pgen-0030027-b009] Lorincz MC, Schubeler D, Goeke SC, Walters M, Groudine M (2000). Dynamic analysis of proviral induction and De Novo methylation: Implications for a histone deacetylase-independent, methylation density-dependent mechanism of transcriptional repression. Mol Cell Biol.

[pgen-0030027-b010] Keshet I, Yisraeli J, Cedar H (1985). Effect of regional DNA methylation on gene expression. Proc Natl Acad Sci U S A.

[pgen-0030027-b011] Schubeler D, Lorincz MC, Cimbora DM, Telling A, Feng YQ (2000). Genomic targeting of methylated DNA: Influence of methylation on transcription, replication, chromatin structure, and histone acetylation. Mol Cell Biol.

[pgen-0030027-b012] Hsieh CL (1997). Stability of patch methylation and its impact in regions of transcriptional initiation and elongation. Mol Cell Biol.

[pgen-0030027-b013] Yin H, Blanchard KL (2000). DNA methylation represses the expression of the human erythropoietin gene by two different mechanisms. Blood.

[pgen-0030027-b014] Kass SU, Goddard JP, Adams RL (1993). Inactive chromatin spreads from a focus of methylation. Mol Cell Biol.

[pgen-0030027-b015] Graessmann A, Sandberg G, Guhl E, Graessmann M (1994). Methylation of single sites within the herpes simplex virus tk coding region and the simian virus 40 T-antigen intron causes gene inactivation. Mol Cell Biol.

[pgen-0030027-b016] Curradi M, Izzo A, Badaracco G, Landsberger N (2002). Molecular mechanisms of gene silencing mediated by DNA methylation. Mol Cell Biol.

[pgen-0030027-b017] Feng YQ, Seibler J, Alami R, Eisen A, Westerman KA (1999). Site-specific chromosomal integration in mammalian cells: Highly efficient CRE recombinase-mediated cassette exchange. J Mol Biol.

[pgen-0030027-b018] Schubeler D, Lorincz MC, Groudine M (2001). Targeting silence: The use of site-specific recombination to introduce in vitro methylated DNA into the genome. Sci STKE.

[pgen-0030027-b019] Lorincz MC, Dickerson DR, Schmitt M, Groudine M (2004). Intragenic DNA methylation alters chromatin structure and elongation efficiency in mammalian cells. Nat Struct Mol Biol.

[pgen-0030027-b020] Wang H, Zhang Y, Cheng Y, Zhou Y, King DC (2006). Experimental validation of predicted mammalian erythroid cis-regulatory modules. Genome Res.

[pgen-0030027-b021] Welch JJ, Watts JA, Vakoc CR, Yao Y, Wang H (2004). Global regulation of erythroid gene expression by transcription factor GATA-1. Blood.

[pgen-0030027-b022] Schneider R, Bannister AJ, Myers FA, Thorne AW, Crane-Robinson C (2003). Histone H3 lysine 4 methylation patterns in higher eukaryotic genes. Nat Cell Biol.

[pgen-0030027-b023] Martens JH, Verlaan M, Kalkhoven E, Zantema A (2003). Cascade of distinct histone modifications during collagenase gene activation. Mol Cell Biol.

[pgen-0030027-b024] Hazzalin CA, Mahadevan LC (2005). Dynamic acetylation of all lysine 4-methylated histone H3 in the mouse nucleus: Analysis at c-fos and c-jun. PLoS Biol.

[pgen-0030027-b025] Hong L, Schroth GP, Matthews HR, Yau P, Bradbury EM (1993). Studies of the DNA binding properties of histone H4 amino terminus. Thermal denaturation studies reveal that acetylation markedly reduces the binding constant of the H4 “tail” to DNA. J Biol Chem.

[pgen-0030027-b026] Lee DY, Hayes JJ, Pruss D, Wolffe AP (1993). A positive role for histone acetylation in transcription factor access to nucleosomal DNA. Cell.

[pgen-0030027-b027] Tse C, Sera T, Wolffe AP, Hansen JC (1998). Disruption of higher-order folding by core histone acetylation dramatically enhances transcription of nucleosomal arrays by RNA polymerase III. Mol Cell Biol.

[pgen-0030027-b028] Imbalzano AN, Kwon H, Green MR, Kingston RE (1994). Facilitated binding of TATA-binding protein to nucleosomal DNA. Nature.

[pgen-0030027-b029] Sewack GF, Ellis TW, Hansen U (2001). Binding of TATA binding protein to a naturally positioned nucleosome is facilitated by histone acetylation. Mol Cell Biol.

[pgen-0030027-b030] Metivier R, Penot G, Hubner MR, Reid G, Brand H (2003). Estrogen receptor-alpha directs ordered, cyclical, and combinatorial recruitment of cofactors on a natural target promoter. Cell.

[pgen-0030027-b031] Hassan AH, Prochasson P, Neely KE, Galasinski SC, Chandy M (2002). Function and selectivity of bromodomains in anchoring chromatin-modifying complexes to promoter nucleosomes. Cell.

[pgen-0030027-b032] Agalioti T, Lomvardas S, Parekh B, Yie J, Maniatis T (2000). Ordered recruitment of chromatin modifying and general transcription factors to the IFN-beta promoter. Cell.

[pgen-0030027-b033] Agalioti T, Chen G, Thanos D (2002). Deciphering the transcriptional histone acetylation code for a human gene. Cell.

[pgen-0030027-b034] Xu W, Edmondson DG, Roth SY (1998). Mammalian GCN5 and P/CAF acetyltransferases have homologous amino-terminal domains important for recognition of nucleosomal substrates. Mol Cell Biol.

[pgen-0030027-b035] Schiltz RL, Mizzen CA, Vassilev A, Cook RG, Allis CD (1999). Overlapping but distinct patterns of histone acetylation by the human coactivators p300 and PCAF within nucleosomal substrates. J Biol Chem.

[pgen-0030027-b036] Lehnertz B, Ueda Y, Derijck AA, Braunschweig U, Perez-Burgos L (2003). Suv39h-mediated histone H3 lysine 9 methylation directs DNA methylation to major satellite repeats at pericentric heterochromatin. Curr Biol.

[pgen-0030027-b037] Nguyen CT, Weisenberger DJ, Velicescu M, Gonzales FA, Lin JC (2002). Histone H3-lysine 9 methylation is associated with aberrant gene silencing in cancer cells and is rapidly reversed by 5-aza-2′-deoxycytidine. Cancer Res.

[pgen-0030027-b038] Sarraf SA, Stancheva I (2004). Methyl-CpG binding protein MBD1 couples histone H3 methylation at lysine 9 by SETDB1 to DNA replication and chromatin assembly. Mol Cell.

[pgen-0030027-b039] Feng YQ, Desprat R, Fu H, Olivier E, Lin CM (2006). DNA methylation supports intrinsic epigenetic memory in mammalian cells. PLoS Genet.

[pgen-0030027-b040] Vakoc CR, Mandat SA, Olenchock BA, Blobel GA (2005). Histone H3 lysine 9 methylation and HP1gamma are associated with transcription elongation through mammalian chromatin. Mol Cell.

[pgen-0030027-b041] Thompson T, Fan H (1985). Mapping of DNase I-hypersensitive sites in the 5′ and 3′ long terminal repeats of integrated moloney murine leukemia virus proviral DNA. Mol Cell Biol.

[pgen-0030027-b042] Lorincz MC, Schubeler D, Groudine M (2001). Methylation-mediated proviral silencing is associated with MeCP2 recruitment and localized histone H3 deacetylation. Mol Cell Biol.

[pgen-0030027-b043] Fatemi M, Pao MM, Jeong S, Gal-Yam EN, Egger G (2005). Footprinting of mammalian promoters: Use of a CpG DNA methyltransferase revealing nucleosome positions at a single molecule level. Nucleic Acids Res.

[pgen-0030027-b044] Gal-Yam EN, Jeong S, Tanay A, Egger G, Lee AS (2006). Constitutive nucleosome depletion and ordered factor assembly at the GRP78 promoter revealed by single molecule footprinting. PLoS Genet.

[pgen-0030027-b045] Hisano M, Ohta H, Nishimune Y, Nozaki M (2003). Methylation of CpG dinucleotides in the open reading frame of a testicular germ cell-specific intronless gene, Tact1/Actl7b, represses its expression in somatic cells. Nucleic Acids Res.

[pgen-0030027-b046] Roh TY, Cuddapah S, Zhao K (2005). Active chromatin domains are defined by acetylation islands revealed by genome-wide mapping. Genes Dev.

[pgen-0030027-b047] Dilworth FJ, Fromental-Ramain C, Yamamoto K, Chambon P (2000). ATP-driven chromatin remodeling activity and histone acetyltransferases act sequentially during transactivation by RAR/RXR In vitro. Mol Cell.

[pgen-0030027-b048] Lu H, Pise-Masison CA, Fletcher TM, Schiltz RL, Nagaich AK (2002). Acetylation of nucleosomal histones by p300 facilitates transcription from tax-responsive human T-cell leukemia virus type 1 chromatin template. Mol Cell Biol.

[pgen-0030027-b049] Kim TH, Barrera LO, Zheng M, Qu C, Singer MA (2005). A high-resolution map of active promoters in the human genome. Nature.

[pgen-0030027-b050] Burch JB, Weintraub H (1983). Temporal order of chromatin structural changes associated with activation of the major chicken vitellogenin gene. Cell.

[pgen-0030027-b051] Litt MD, Hansen RS, Hornstra IK, Gartler SM, Yang TP (1997). 5-Azadeoxycytidine-induced chromatin remodeling of the inactive X-linked HPRT gene promoter occurs prior to transcription factor binding and gene reactivation. J Biol Chem.

[pgen-0030027-b052] Martinez-Balbas MA, Dey A, Rabindran SK, Ozato K, Wu C (1995). Displacement of sequence-specific transcription factors from mitotic chromatin. Cell.

[pgen-0030027-b053] Mizutani T, Ito T, Nishina M, Yamamichi N, Watanabe A (2002). Maintenance of integrated proviral gene expression requires Brm, a catalytic subunit of SWI/SNF complex. J Biol Chem.

[pgen-0030027-b054] Frigola J, Song J, Stirzaker C, Hinshelwood RA, Peinado MA (2006). Epigenetic remodeling in colorectal cancer results in coordinate gene suppression across an entire chromosome band. Nat Genet.

[pgen-0030027-b055] Macleod D, Charlton J, Mullins J, Bird AP (1994). Sp1 sites in the mouse aprt gene promoter are required to prevent methylation of the CpG island. Genes Dev.

[pgen-0030027-b056] Bird A, Taggart M, Frommer M, Miller OJ, Macleod D (1985). A fraction of the mouse genome that is derived from islands of nonmethylated, CpG-rich DNA. Cell.

[pgen-0030027-b057] Forrester WC, Epner E, Driscoll MC, Enver T, Brice M (1990). A deletion of the human beta-globin locus activation region causes a major alteration in chromatin structure and replication across the entire beta-globin locus. Genes Dev.

[pgen-0030027-b058] Shang Y, Hu X, DiRenzo J, Lazar MA, Brown M (2000). Cofactor dynamics and sufficiency in estrogen receptor-regulated transcription. Cell.

[pgen-0030027-b059] Edmondson DG, Davie JK, Zhou J, Mirnikjoo B, Tatchell K (2002). Site-specific loss of acetylation upon phosphorylation of histone H3. J Biol Chem.

[pgen-0030027-b060] Sawado T, Halow J, Bender MA, Groudine M (2003). The beta-globin locus control region (LCR) functions primarily by enhancing the transition from transcription initiation to elongation. Genes Dev.

