# Supplementary material for: An Unmethylated 3′ Promoter-Proximal Region Is Required for Efficient Transcription Initiation
Source: PLoS Genet. 2007 Feb 16;3(2):e27. doi: 10.1371/journal.pgen.0030027 (PMC1797817; doi:10.1371/journal.pgen.0030027)
Supplement: Supplementary file 1 [file pgen.0030027.sg001.pdf]

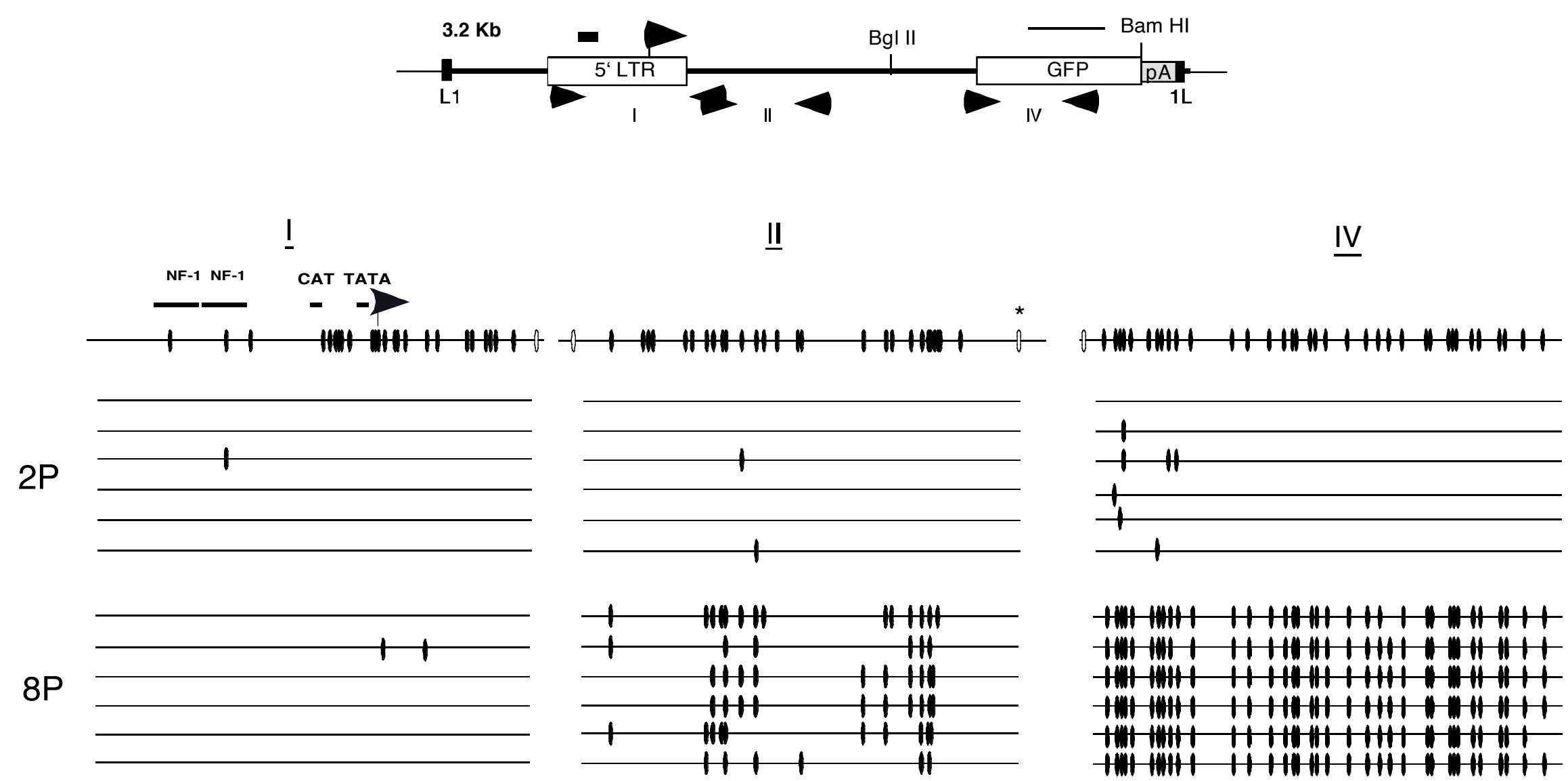

Figure S1
