# Supplementary material for: An Unmethylated 3′ Promoter-Proximal Region Is Required for Efficient Transcription Initiation
Source: PLoS Genet. 2007 Feb 16;3(2):e27. doi: 10.1371/journal.pgen.0030027 (PMC1797817; doi:10.1371/journal.pgen.0030027)
Supplement: Supplementary file 2 [file pgen.0030027.sg002.pdf]

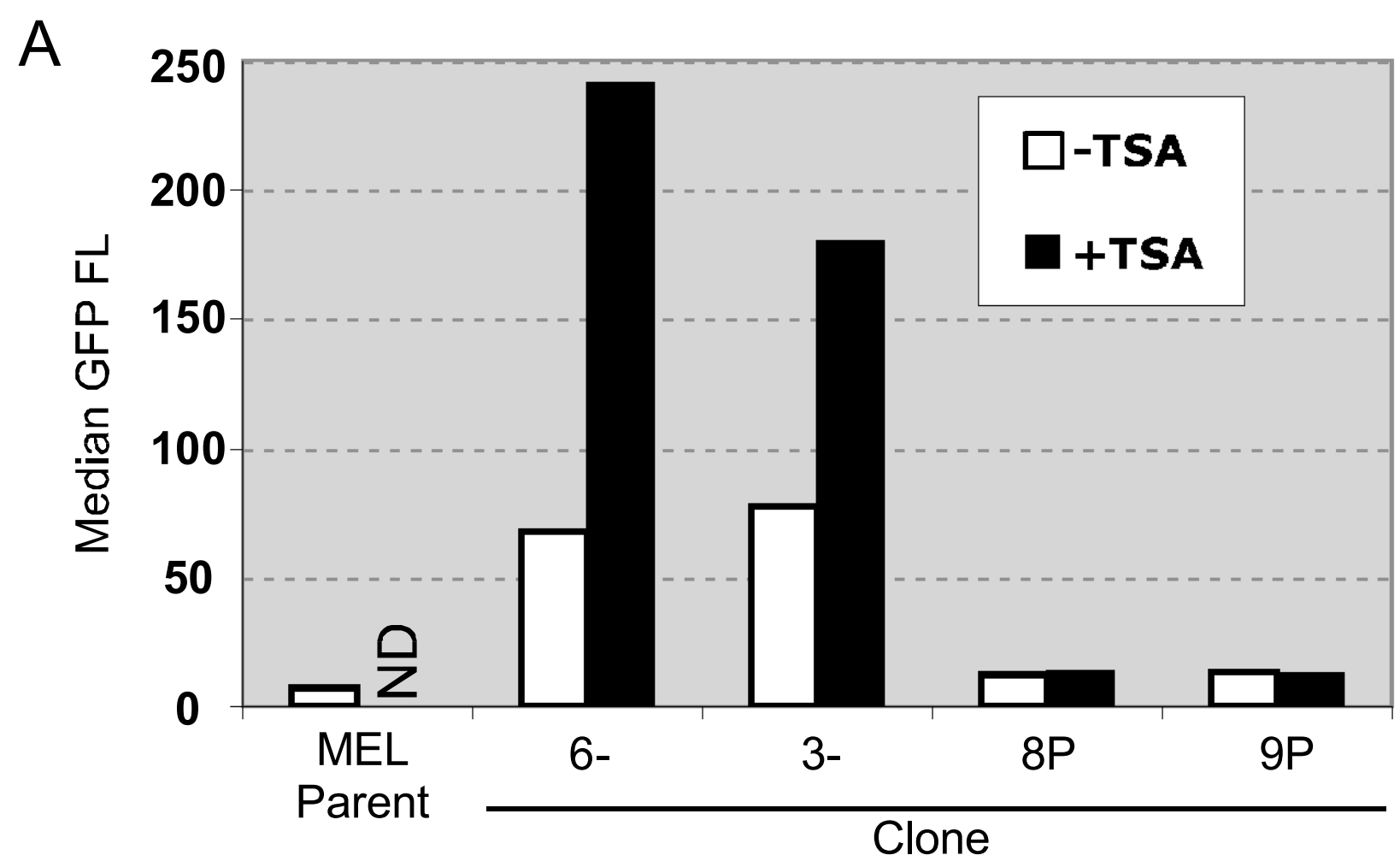
A
Median GFP FL
250
200
150
100
50
0
-TSA
+TSA
ND
MEL
Parent
6-
3-
8P
9P
Clone

Figure S2
