# Supplementary material for: An Unmethylated 3′ Promoter-Proximal Region Is Required for Efficient Transcription Initiation
Source: PLoS Genet. 2007 Feb 16;3(2):e27. doi: 10.1371/journal.pgen.0030027 (PMC1797817; doi:10.1371/journal.pgen.0030027)
Supplement: Supplementary file 3 [file pgen.0030027.sg003.pdf]

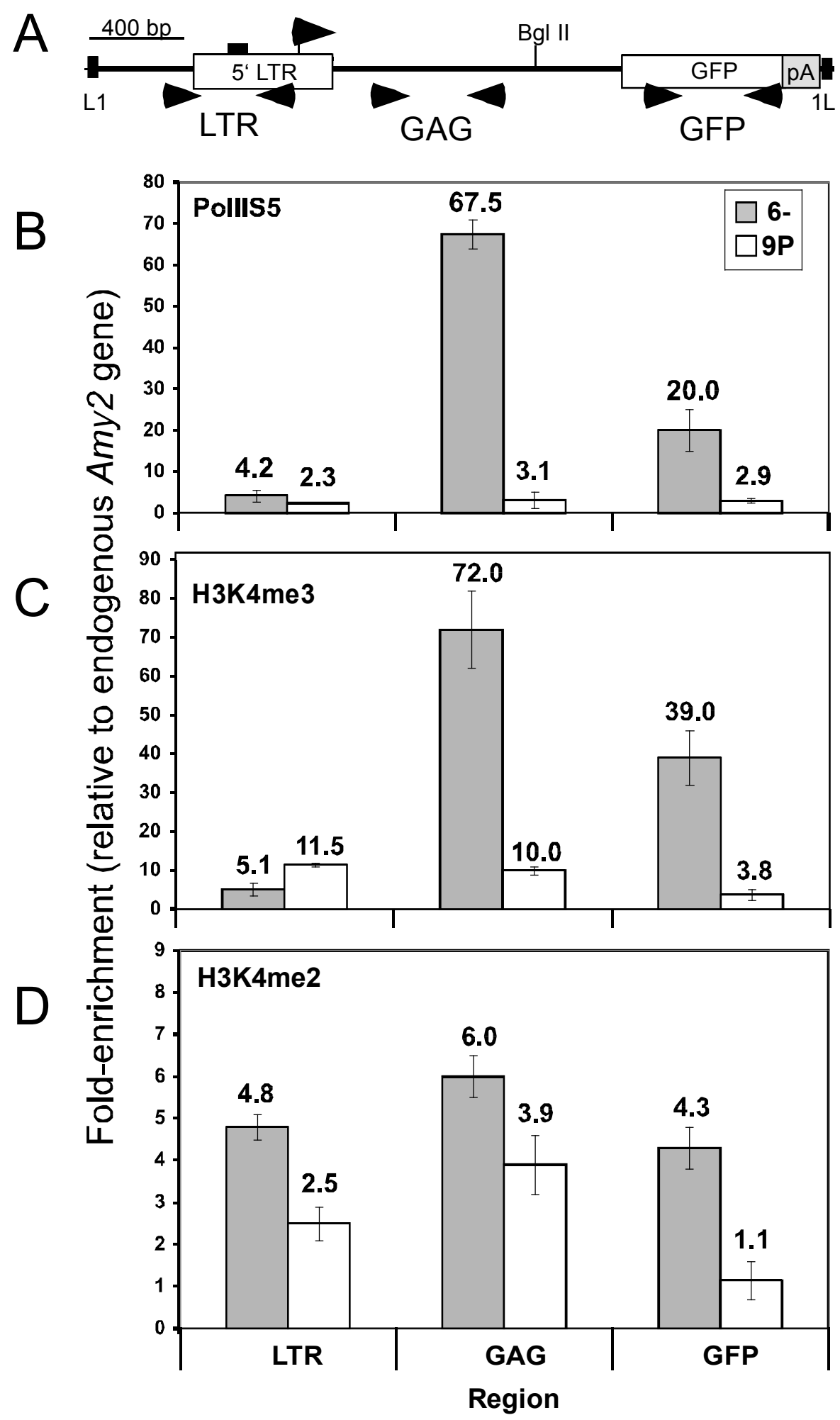
A
400 bp
Bgl II
5' LTR
GFP
pA
L1
1L
LTR
GAG
GFP
B
PolIIS5
6-
9P
4.2
2.3
67.5
3.1
20.0
2.9
C
H3K4me3
5.1
11.5
72.0
10.0
39.0
3.8
D
H3K4me2
4.8
2.5
6.0
3.9
4.3
1.1
Fold-enrichment (relative to endogenous Amy2 gene)
LTR
GAG
GFP
Region

Figure S3
