# Supplementary material for: An Unmethylated 3′ Promoter-Proximal Region Is Required for Efficient Transcription Initiation
Source: PLoS Genet. 2007 Feb 16;3(2):e27. doi: 10.1371/journal.pgen.0030027 (PMC1797817; doi:10.1371/journal.pgen.0030027)
Supplement: Supplementary file 4 [file pgen.0030027.st001.doc]

**Table S1.** Primers used in this study

| RT-PCR | | |
| --- | --- | --- |
| **Transgene** LTR + strand  GFP - strand | tctgagtgattgactacccgtc agggcacgggcagcttgc | |
| ***β-actin*** + strand  **- strand** | tcatgaagtgtgacgttgacatccgt  cctagaagcacttgcggtgcacgatggag | |
| **Bisulfite analysis** | | |
| **LTR (I)** 1^st^ round: +  -  2^nd^ round: +  - | TAGGTTTGGTAAGTTAGTTTAAGTAAYGTT TAAAAAAATAATAACAATCTAACCCRAAC  TTGTAAGGTATGGAAAAATATATAATTG TAAATTACTAACCAACTTACCTCCCRATAA | |
| **GAG (II)** 1^st^ round: +  -  2^nd^ round: +  - | TAGGTTTGGTAAGTTAGTTTAAGTAAYGTT TAAAAAAATAATAACAATCTAACCCRAAC  GATTTATTATTGGGAGGTAAGTTGGTTAGT TAAAAAAATAATAACAATCTAACCCRAAC | |
| **GFP (IV)** 1^st^ round: +  -  2^nd^ round: +  - | ATTATTTTCTAGATTGTTATGGTGAGTAAGGG CTCAAGCTTATAATTATACTCCAACTTATACCCCA GAGGAGTTGTTTATYGGGGTGGTGTTT  CTCAAGCTTATAATTATACTCCAACTTATACC | |
| ChIP analysis (duplex PCR) | | |
| **LTR** +  - | | catgtgaaagaccccacctgtag tggttctctagaaactgctga |
| **GAG** +  - | | cgtttttgtggcccgacctg TCAAACTTAAGGGAGTGGTAACAGGC |
| **GFP** +  - | | gacaccctggtgaaccgcatcg TGCTCAGGTAGTGGTTGTC |
| ***Amy2*** +  -  - | | TCAGTTGTAATTCTCCTTGTACGG CCTCCCATCTGAAGTATGTGGGTC  CATTCCTTGGCAATATCAACC |
| **ChIP analysis (real-time PCR)** | | |
| ***β-maj*** +  - | | ctgctcacacaggatagagaggg  gcaaatgtgaggagcaactgatc |
| ***Gnas*** +  - | | CAACGAGCCTGCCCAGGGAGACCATAG  CTGTAGTCTTCTCGGAAGCCAGCG |
| **TATA** +  - | | AACCATCAGATGTTTCCAGGGTG AGTCGGATGCAACTGCAAGAGGG |
| **GFP** +  - | | ACTACAACAGCCACAACGTCTATATCA GGCGGATCTTGAAGTTCACC |

Y= C or T	Underlined Sequence = HpaII site

R= A or G
